# Genome Sequence of Bovine Viral Diarrhea Virus Strain 10JJ-SKR, Belonging to Genotype 1d

**DOI:** 10.1128/genomeA.00565-13

**Published:** 2013-08-08

**Authors:** Soo-Kyung Joo, Seong-In Lim, Hye-Young Jeoung, Jae-Young Song, Jae-Ku Oem, Seong-Hwan Mun, Dong-Jun An

**Affiliations:** Animal and Plant Quarantine Agency, Anyang, Republic of Koreaa; Jeju Veterinary Research Institute, Jeju, Republic of Koreab

## Abstract

Here, we report the complete genome sequence of a bovine viral diarrhea virus (BVDV) belonging to genotype 1d, strain 10JJ-SKR, which was isolated from cattle. The complete genome is 12,267 nucleotides (nt) in length, with a single large open reading frame. This is the first report of a BVDV belonging to genotype 1d and will enable further study of the molecular and epidemiological characteristics of this virus.

## GENOME ANNOUNCEMENT

Bovine viral diarrhea virus (BVDV) is common in cattle and causes significant economic losses to the livestock industry (1–3). BVDV, together with classical swine fever virus and border disease virus, belongs to the genus *Pestivirus* within the family *Flaviviridae*. BVDV has been classified into two species, BVDV-1 and BVDV-2 (4). BVDV-1 has at least 15 subtypes (5), and BVDV-2 has 2 subtypes (6). The complete genome sequences of BVDV-1a, -1b, -1e, -1m, -2a, and -2b have been reported. Here, we report the complete genome sequence of a novel BVDV strain, 10JJ-SKR, belonging to the genotype 1d.

Strain 10JJ-SKR was isolated from the blood of a cow on Jeju island, South Korea, in 2010. Total viral RNA was extracted from infected Madin-Darby bovine kidney (MDBK) epithelial cells using an RNeasy minikit (Qiagen; catalog no. 74104). cDNA was obtained by reverse transcription-PCR with Superscript III reverse transcriptase (Invitrogen). Ten sets of primers were designed based on the conserved sequences of other BVDVs (accession numbers M96751, U63479, and M96687) from the GenBank database at NCBI. The 5′ and 3′ ends of the viral genome were amplified by rapid amplification of cDNA ends (Seegene; catalog no. K2000). All fragments were sequenced in both directions and the sequences were aligned using ClustalW 1.83 (7). A phylogenetic tree was then constructed in Mega 4.1 using the neighbor-joining method.

The complete genome of strain 10JJ-SKR comprises 12,267 nucleotides (nt), including a 384-nt 5′ untranslated region (UTR) and a 192-nt 3′ UTR. The open reading frame encodes a polyprotein of 3,897 amino acids (aa). The structural proteins encoded by strain JJ10-SKR contain 14 potential N-linked glycosylation sites. The nt sequence of the E2 gene fragment from strain JJ10-SKR was 76.5%, 70.7%, 91.7%, 75.1%, 73.6%, 65.2%, and 65.8% homologous with those of strains Oregon C24V (1a), CP7 (1b), SH9 (1d), KS86-1ncp (1e), ZM-95 (1m), and 890 (2a) and Hokudai-Lab/09 (2b), respectively; the respective aa sequence homologies were 77.9%, 77.1%, 91.4%, 78.6%, 78.6%, 63.6%, and 65.0%. A phylogenetic tree was constructed based on all BVDV-1d partial sequences entered into the GenBank database. Strain 10JJ-SKR clustered within subgroup BVDV 1d.

This is the first report of the identification of a complete BVDV 1d genome sequence. This sequence will form the basis of further studies to examine the molecular characteristics of this virus strain.

### Nucleotide sequence accession number.

The complete genome sequence of BVDV strain 10JJ-SKR was deposited in GenBank under accession no. KC757383.
